# ASCO update on lymphoma

**DOI:** 10.1007/s12254-017-0372-y

**Published:** 2017-11-24

**Authors:** Michael A. Fridrik

**Affiliations:** Department of Internal Medicine 3, Centre for Hematology and Medical Oncology, Kepler University Hospital, Krankenhausstraße 9, 4021 Linz, Austria

**Keywords:** Indolent, Aggressive lymphoma, Multiple myeloma, Treatment

## Abstract

Abstracts concerning indolent and aggressive lymphoma and multiple myeloma with clinical relevance from the ASCO 2017 meeting are discussed.

## Introduction

The annual meeting of the American Society of Clinical Oncology is the most important meeting in this field. This year 735 abstracts containing lymphoma were presented. I have tried to find the abstracts with most relevance to our clinical decisions for our patients.

## Indolent lymphoma

Finn et al. (abstract #7005) presented long-term results of the BRIGHT study. Treatment-naïve patients with indolent B‑cell lymphoma or mantle-cell lymphoma were assigned by the participating centre to a standard treatment with 6–8 cycles rituximab, cyclophosphamide, doxorubicin, vincristine, prednisone (R-CHOP) or rituximab, cyclophosphamide, vincristine, prednisone (R-CVP). These patients were then randomised to the assigned standard treatment or rituximab bendamustine (R-Benda). The study was published in *Blood* in 2014 and could demonstrate noninferiority for R‑Benda. In this paper the 5‑year progression-free survival (PS), event-free survival (EFS) and duration of response were superior, but not significantly different. Updated 5‑year results were presented. The 5‑year PFS rate was 65.5% (95% CI 58.5–71.6) and 55.8% (48.4–62.5), and OS was 81.7% (75.7–86.3) and 85% (79.3–89.3) for R-Benda and R‑CHOP/R-CVP, respectively. Most importantly, they did not observe an increased death rate due to infection in the R‑Benda arm. However, there was an increased number of secondary malignancies due to nonmelanoma skin cancer in the R‑Benda arm.

In abstract #7501 Rummel et al. presented the long-term outcome of the StiL NHL 1‑2003 study [1]. They randomised untreated indolent B‑cell and mantle-cell lymphomas to R‑CHOP and R‑Benda. Despite a significant better hazard ratio (HR) of 0.55 in favour of R‑Benda the 10-year overall survival was not different (71% for R‑Benda and 66% for R‑CHOP). They did not observe an increased infection rate in the R‑Benda arm.

What can we learn from these two ASCO abstracts? In the StiL trial patients received no maintenance treatment and PFS was significant better in the R‑Benda arm. In all, 45% of patients in the BRIGHT trial received rituximab maintenance and the R‑Benda patients had a nonsignificant benefit in PFS. At the 2016 ASH meeting Marcus et al. presented the first results of the GALLIUM trial. In this trial untreated indolent B‑cell lymphomas were randomised to chemotherapy with rituximab and rituximab maintenance versus obinutuzumab with chemotherapy followed by obinutuzumab maintenance. The patients with bendamustine in this trial had no difference in PFS. They described a higher infection rate during the maintenance treatment after obinutuzumab bendamustine compared to the patients with CHOP or CVP chemotherapy. All patients in the GALLIUM trial received rituximab or obinutuzumab maintenance and PFS was not different. Rituximab maintenance may improve the inferior results of R‑CHOP or R‑CVP compared to R‑Benda. After R‑Benda rituximab maintenance may not be needed in previously untreated indolent lymphoma. Unfortunately the StiL trial NHL7-2008 did not randomise rituximab maintenance versus no maintenance after R‑Benda, but 2 versus 4 years of rituximab maintenance, so this question will not be answered. R‑Benda is not registered as first-line therapy for indolent B‑cell lymphoma in the European Union. In March 2017 Mundipharm Austria sent safety information about reactivation of hepatitis B, serious and fatal infections and a prolonged T‑helper lymphopenia occurring after R‑Benda. The infection rate could not be confirmed in these two studies. To what extent rituximab maintenance treatment could be responsible for the increased infection rate is not clear. Combined analyses of all R‑Benda studies with and without maintenance would be of interest.

The MAGNIFY trial treated patients with indolent B‑cell lymphoma relapsing or refractory to one or more prior therapies with a chemotherapy-free regimen with 12 cycles of lenalidomide 20 mg every day for 21 days combined with rituximab 375 mg/m^2^ on day one of a 28-day cycle (R2). All patients not progressing at the end of treatment were randomised to rituximab ± lenalidomide maintenance until progression. This chemotherapy-free regimen had impressive remission rates of 61% and 1‑year PFS of 66% (abstract #7502). The RELEVANCE study is randomising R2 versus R‑chemotherapy and will answer the question whether R2 is superior to R‑chemotherapy.

## Diffuse large B‑cell lymphoma (DLBCL)

Bulky DLBCL have an inferior survival compared to nonbulky disease. However, bulky is not uniformly defined. A maximal diameter of ≥5 cm, ≥7.5 cm and ≥10 cm are used. Usually radiotherapy to residual disease or bulky disease is recommended. However, data are conflicting. In their UNFOLDER trail the German High-Grade Non-Hodgkin Lymphoma Study Group (DSHNHL) randomised younger DLBCL patients to R‑CHOP 21 vs. R‑CHOP14 and ± radiotherapy at the end of treatment to bulky disease at diagnosis. The randomisation was terminated early because of a significant shorter PFS for patients in the no radiotherapy arm. They also demonstrated a PFS benefit for radiotherapy on initial bulky disease in elderly patients. In the ASCO abstract #7506 Michael Pfreundschuh presented for the DSHNHL a comparison of patients with bulky (≥7.5 cm) in the OPTIMAL >60 patients with bulky disease in the RICOVER-60 trial. In the OPTIMAL >60 trial bulky sites were irradiated if they were PET positive after the end of their chemoimmunotherapy. In the RIVOVER-60 trial all patients with bulky disease at diagnosis had to be radiated at the end of their chemoimmunotherapy. The 2‑year PFS and OS in OPTIMAL >60 was 79% and 88%, respectively, compared to 75% and 78% of the RICOVER-60 patients. In a multivariable analysis adjusting for the IPI risk factors, the HR of the OPTIMAL >60 compared to the RICOVER-60 bulk patients was 0.7 (95% CI: 03.; 1.5; *p* = 0.345) for PFS and 0.5 (95% CI: 02.; 1.3; *p* = 0.154) for OS. According to these data, patients >60 years with DLBCL and bulky disease can be safely spared from radiotherapy if they become PET negative after chemoimmunotherapy. However, it is still unclear if all PET positive patients need radiotherapy.

## Multiple myeloma (MM)

Bisphosphonate therapy is a standard supportive therapy for patients with MM. However treatment is cumbersome because of side effects and dose adjustments to renal function. Denosumab is a monoclonal antibody inhibiting the receptor activator of NF-κB (RANK) ligand. Raje et al. reported in the abstract #8005 of a randomised study in MM patients. In this placebo-controlled study zoledronic acid was compared to denosumab. In 1718 randomised patients noninferiority could be demonstrated with respect to the first skeletal event. There were significantly fewer adverse events in the denosumab arm. A cost-effective analysis was not presented.

### Take-home message

R-Benda is as least as effective as R‑CHOP in indolent B‑cell lymphoma. However, conflicting data regarding infectious complications exist. After R‑Benda R‑maintenance treatment may not be needed in previously untreated indolent lymphoma. DLBCL with bulky disease can be spared from radiotherapy if PET is negative after RCHOP.

Denosumab is equally effective as zoledronic acid in MM, but less toxic.
